# Apolipoprotein E4 Alters Astrocyte Fatty Acid Metabolism and Lipid Droplet Formation

**DOI:** 10.3390/cells8020182

**Published:** 2019-02-20

**Authors:** Brandon C. Farmer, Jude Kluemper, Lance A. Johnson

**Affiliations:** 1 Department of Physiology, University of Kentucky, 800 Rose Street, Rm: MS-609, Lexington, KY 40536, USA; brandon.c.farmer@uky.edu (B.C.F.); jude.kluemper@uky.edu (J.K.); 2 Sanders-Brown Center on Aging, University of Kentucky, 800 Rose Street, Lexington, KY 40536, USA

**Keywords:** APOE, astrocytes, lipid droplets, lipid metabolism, AD, PLIN-2, fatty acid oxidation

## Abstract

Lipid droplets (LDs) serve as energy rich reservoirs and have been associated with apolipoprotein E (*APOE*) and neurodegeneration. The E4 allele of *APOE* (E4) is the strongest genetic risk factor for the development of late onset Alzheimer’s disease (AD). Since both E4 carriers and individuals with AD exhibit a state of cerebral lipid dyshomeostasis, we hypothesized that *APOE* may play a role in regulating LD metabolism. We found that astrocytes expressing E4 accumulate significantly more and smaller LDs compared to E3 astrocytes. Accordingly, expression of perilipin-2, an essential LD protein component, was higher in E4 astrocytes. We then probed fatty acid (FA) metabolism and found E4 astrocytes to exhibit decreased uptake of palmitate, and decreased oxidation of exogenously supplied oleate and palmitate. We then measured oxygen consumption rate, and found E4 astrocytes to consume more oxygen for endogenous FA oxidation and accumulate more LD-derived metabolites due to incomplete oxidation. Lastly, we found that E4 astrocytes are more sensitive to carnitine palmitoyltransferase-1 inhibition than E3 astrocytes. These findings offer the potential for further studies investigating the link between astrocyte lipid storage, utilization, and neurodegenerative disease as a function of *APOE* genotype.

## 1. Introduction

Apolipoprotein E (apoE) is associated with circulating lipoproteins, specifically very low-density lipoproteins and high-density lipoproteins [1]. The polymorphic *APOE* gene encodes for three isoforms, E2, E3, and E4, with frequencies of 8%, 77%, and 15%, respectively, in the general US population [2]. E4 is the strongest genetic risk factor for late onset Alzheimer’s disease (AD) [2]. In addition to established effects on AD neuropathology, *APOE* genotype is also recognized as a strong modulator of cerebral metabolism [3,4,5,6]. In fact, cognitively normal E4+ individuals demonstrate alterations in cerebral fatty acid (FA) and carbohydrate metabolism congruent with AD patients [4,7]. Understanding the metabolic effects of apoE in the brain may be a critical step in discovering the underlying mechanism(s) of E4-driven AD risk. 

The brain is one of the largest site of apoE synthesis, second to the liver [8]. The majority of brain apoE is astrocyte derived, though under certain conditions such as trauma, neurons and microglia also synthesize apoE [9]. ApoE binds to receptors of the low-density lipoprotein receptor family, which are expressed on astrocytes as well as neurons [10]. Astrocytes aid in shuttling lipids from the blood-brain barrier (BBB) to neurons by both binding and internalizing BBB permeable FAs from the endothelial cells, and loading lipid-free apoE with cargo through action of the ATP-binding cassette transporters such as ABCA-1 [11,12]. Therefore, astrocytes are key players in the lipid uptake-apoE-lipidation axis, and are vital to maintaining brain lipid homeostasis and proper neuronal function. Not only are astrocytes critical for uptake and export of FAs and lipid-loaded lipoproteins, they also are the main cell population mediating β-oxidation of FAs in the brain [13,14]. Astrocytes contain a higher relative density of mitochondria than the surrounding neuropil, giving them superior machinery to oxidize FA [15]. Furthermore, carnitine palmitoyltransferase-1 (CPT-1), an essential enzyme for the beta-oxidation of long chain FA, is preferentially expressed in astrocytes compared to neurons, microglia, and oligodendrocytes [16]. Recent studies clearly show that the brain does in fact utilize a significant amount of FAs, with some reports estimating FAs are responsible for up to 20% of cerebral ATP generation [14,17].

Lipid droplets (LD) are organelles containing triacylglycerols and cholesterol esters surrounded by a layer of amphipathic lipids and associated proteins. Once thought to be inert cellular depots of fat, lipid droplets are now considered to be dynamic organelles that play a role in various metabolic diseases [18,19]. Indeed, the overaccumulation of LDs has been linked to atherosclerosis, metabolic syndrome, cancer, and diabetes [20,21]. However, only recently have studies begun to address the potential pathological consequences of abnormal LD accumulation in the brain [18]. Although low amounts of LD are observed in the brain under normal conditions [22], studies have noted increased LD content in neurodegenerative conditions such as Parkinson’s [23] and AD [24,25]. Interestingly, Alois Alzheimer noted glial lipid accumulation in his first description of AD pathology [26], but this has gone relatively unstudied apart from a few reports linking LDs and AD [24,27]. Recent findings suggest that glial cells within the brain form LD as a function of neuronal stress, and in the fly brain this process is apoE dependent [27]. Astrocyte derived E4 has been shown to promote LD formation in fibroblasts [28], and iPSC-derived astrocytes expressing E4 exhibit increased lipid storage in the form of cholesterol [29]. E4 astrocytes also show varied expression of numerous genes associated with lipid metabolism and transport, compared to those expressing E3 [29]. However, apoE-isoform specific modulation of LD accumulation and FA metabolism in astrocytes has not been previously described.

In this study, we characterize an increase in lipid droplet formation and endogenous FA utilization in E4 astrocytes, as well as decreased uptake and oxidation of exogenous FAs. These lipid metabolism events influenced by *APOE* offer both targets for future late onset AD therapies and potential for more studies analyzing the metabolic effects of *APOE* in the brain. 

## 2. Materials and Methods

### 2.1. Cell Culture

E3 and E4 expressing astrocytes were derived from targeted replacement mice expressing human *APOE3* or *APOE4* (kind gift from Dr. David Holtzman). These cell lines secrete apoE in high density lipoprotein-like particles at equivalent levels to primary astrocytes from targeted replacement APOE knock-in mice and have been relied upon for studies of APOE’s role in astrocyte metabolism by many groups [30,31,32,33]. Cells were maintained in Advanced Dulbecco’s Modified Eagle Medium (DMEM) (Gibco, Carlsbad, CA, USA) supplemented with 1 mM sodium pyruvate, 1× Geneticin, and 10% fetal bovine serum. For imaging experiments, cells were grown on sterilized coverslips in 6-well plates pre-treated with poly-L. For lipid loading experiments, 250 µM oleate pre-conjugated to bovine serum albumin (BSA) (Sigma, St. Louis, MO, USA) was added to the media 24 h prior for lipid droplet induction as previously described [34]. 

### 2.2. Western Blotting

Cells were lysed on ice using radioimmunoprecipitation assay Buffer (Thermo Fisher Scientific, Waltham, MA, USA) with 1× proteinase inhibitor (Sigma, St. Louis, MO, USA ). Protein concentration was determined by Pierce bicinchoninic acid (BCA) protein assay (Thermo Fisher Scientific, Waltham, MA, USA). 20–30 µg of protein was diluted with 2× Laemmli Sample Buffer (Bio-Rad Laboratories, Hercules, CA, USA) and heated at 100 °C for 10 min. Protein samples were then loaded on 4–20% PROTEAN TGX gels (Bio-Rad Laboratories, Hercules, CA, USA) for SDS-PAGE. Gels were transferred using a Trans-Blot Turbo Transfer system (Bio-Rad Laboratories, Hercules, CA, USA) onto polyvinylidene fluoride membrane. Membranes were then blocked for 2 h in 1% casein and then incubated overnight in primary antibody solution (1:1000 PLIN-2 (Novus Biologicals, Centennial, CO, USA); 1:1000 APOE (Abcam Inc., Cambridge, MA, USA); 1:1000 β-actin (Novus)) at 4 °C. Membranes were then washed three times for five minutes each with PBS, pH 7.4, 0.05% Tween-20, and then incubated with secondary antibody solution (1:10,000 Goat α rabbit IR 680 (LI-COR, Lincoln, NE, USA); 1:5000 goat α mouse IR 800 (LI-COR)) for 2 h at room temperature. Membranes were then washed as before, with an additional two 10 min washes in PBS. Images were acquired on a LI-COR Odyssey Infrared Scanner. Resulting images were exported to ImageJ for blot densitometry and quantification. 

### 2.3. Lipid Droplet Imaging

After lipid incubation, coverslips were lifted from the wells and cells were fixed for 15 min in 4% PFA, washed 1× with PBS, and incubated for 30 min in 1:1000 LipidSpot (Biotium, Fremont, CA, USA). Coverslips were then mounted onto slides using Fluoroshield mounting media with DAPI (Abcam). Slides were kept at 4 °C. Wide field images were obtained on a Nikon Ti2 microscope using a 20× objective. Single cell images were obtained on a Nikon A1R Laser Scanning Confocal Microscope (Nikon, Tokyo, Japan) using an oil immersed 100× objective. Astrocytes were selected in the 405 channel (DAPI-stained nuclei) and then the 610 channel was added (LipidSpot-stained LDs) and the images were captured. Z-stacks were taken in a range of 10 µM, 40 images per stack. 3-D reconstructions were processed in Imaris 9.2 software using the surfaces module to obtain statistics for lipid droplets. Experimenter was blind to slide identity and slides were processed in random order under identical microscope settings. 

### 2.4. Fatty Acid Uptake Assay

Astrocytes were plated at 50,000 cells/well in CytoStar T plates (Perkin Elmer, Waltham, MA, USA) and allowed to grow to confluency. Media was aspirated, and new glucose free media supplemented with either 0.5 µCi/mL ^14^C-palmitate or ^14^C-oleate (Perkin Elmer) was added to the wells. Scintillation bead technology in the wells emitted light proportionate to the cellular uptake of the FA [35]. Radioactivity counts were read on a Microbeta 2 Microplate Counter (Perkin Elmer) and normalized to protein content in the well using a BCA assay (Thermo Fisher Scientific, Waltham, MA, USA). 

### 2.5. Fatty Acid Oxidation Assay

Astrocytes were plated in a 24-well plate at 300,000 cells/well and allowed to grow to confluency for 24 h. Using a previously published protocol [36], cells were then incubated with 0.5 µCi/mL [1-^14^C] palmitate or 0.5 µCi/mL [1-^14^C] oleate for 24 or 3 h. For the pulse experiment (exogenously supplied FA) radiolabeled FAs were added in nutrient depleted media (Glucose Free DMEM; 5% FBS) for 3 h. Buffered ^14^CO_2_ in the media was then liberated by addition of 1 M hydrochloric acid and captured on a filter paper disc pre-soaked with 1N sodium hydroxide. For the pulse chase experiment (endogenous FA), radiolabeled FAs were added in nutrient rich media (25 mM Glucose; 10% FBS) for 24 h, then aspirated and nutrient depleted media was added to promote oxidation of fatty acid from intracellular lipid pools. Radioactivity of the filter paper was measured in a Microbeta 2 Microplate Counter after addition of 3 mL Ultima-Gold Scintillation Fluid. Acid soluble metabolites, which represent incomplete oxidation of the fatty acids, were extracted from the media and cellular fractions. Total protein from each well was assayed using a BCA assay (Thermo) and all radioactivity measures were normalized to protein content. 

### 2.6. Seahorse Extracellular Flux Analysis

Astrocytes expressing E3 or E4 were seeded in a 96-well microplate at 50,000 cells/well and left to form a monolayer overnight. Media was aspirated and 135 µL of limited nutrient running media (DMEM; 5 mM glucose, 2.5 mM carnitine, 5 mM 4-(2-hydroxyethyl)-1-piperazineethanesulfonic acid) was added to each well and allowed to incubate for 1 h. Then 30 µL of 1 mM sodium palmitate complexed to BSA (6:1 molar ratio) or BSA alone was added to wells according to group assignment, at which point the oxygen consumption rate (OCR) was determined by a Seahorse Extracelluar Flux Analyzer xF96 (Agilent, Santa Clara, CA). At each successive 18 min, an injection of 200 µM etomoxir, 1 µM oligomycin, or 1 µM Rotenone | Antimycin was added to each well to inhibit carnitine palmitoyltransferase-1 (CPT-1), ATP synthetase, or complex I and III respectively. OCR was measured every 6 min. Values were normalized to protein content in each well by a BCA Assay (Thermo). OCR from endogenous FA oxidation was determined by calculating the average etomoxir effect in BSA treated astrocytes (Equation (1)).

(1) $${\bar{OCR}}_{Endo\;FAO}={\bar{OCR}}_{Pre-Eto\;Control}-{\bar{OCR}}_{Post-Eto\;Control}$$

OCR from exogenous FA oxidation was determined by calculating the average difference in basal OCR in the palmitate treated astrocytes versus the basal OCR in BSA treated astrocytes (Equation (2)).

(2) $${\bar{OCR}}_{Exo\;FAO}={\bar{OCR}}_{Pre-Eto\;PA}-{\bar{OCR}}_{Pre-Eto\;Control}$$

### 2.7. Statistical Analysis

All data are expressed as mean  ±  standard error. Comparisons between two groups were analyzed by *t*-test. Multiple groups and/or multiple time points were analyzed using ANOVAs (Prism, Graphpad, San Diego, CA, USA), or repeated measures ANOVA (time  ×  groups) (SPSS). Statistical significance was determined using an error probability level of *p*  < 0.05 corrected by a false discovery rate (FDR) analysis (Benjamini Hochberg method).

## 3. Results

### 3.1. E4 Astrocytes Increased LD Count, Increased Cellular LD Volume, Decreased LD Size

**Figure 1:**
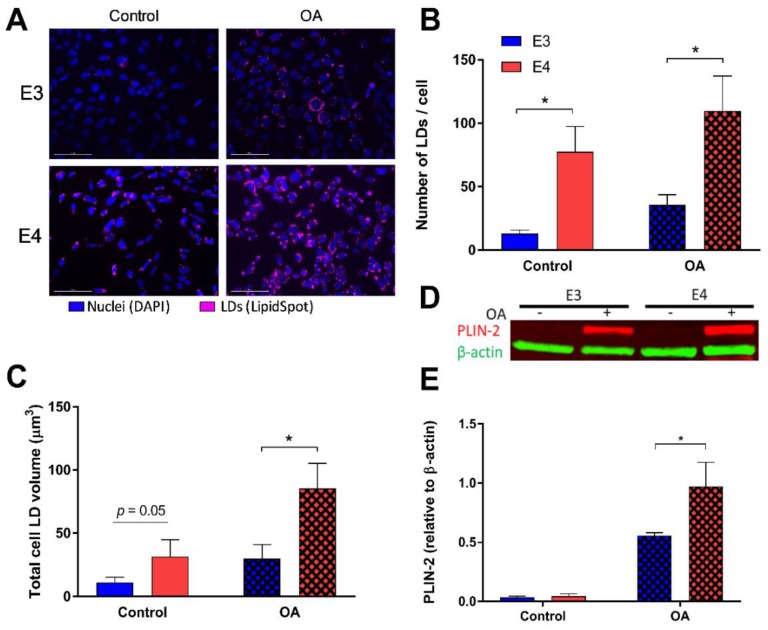
E4 astrocytes have increased lipid droplet (LD) count, LD volume, and LD associated protein. (**A**) Astrocytes expressing either E3 or E4 were stained with LipidSpot to quantify lipid content under control conditions (left), and after 24 h oleic acid (OA) supplementation (“lipid-loaded”; right). The number of LDs per individual cell (**B**) and total LD volume per cell (**C**) were quantified. Values represented are means +/− SEM (*n* = 10). Data were analyzed by *t*-test for specific comparisons between means; * *p* < 0.05. (**D**) Representiative image of the total expression of perilipin-2 (PLIN2) and β-actin in control or lipid-loaded E3 and E4 astrocytes was determined by SDS-PAGE and Western blotting and quantified (**E**). Values represent mean +/− SEM (*n* = 3). Data were analyzed by by *t*-test; * *p* < 0.05. Scale bar = 100 µm.

We first asked if APOE influences lipid storage within astrocytes. To answer this, we lipid-loaded E3 and E4 astrocytes with 250 μM oleate for 24 h, stained for neutral LDs (Figure 1A and Figure S3A), and quantified LDs per cell (Figure 1B and Figure S3B). E4 astrocytes showed a significant increase in the number of LDs, both at basal levels and in a FA-rich environment, compared to E3. (Figure 1B) Total LD volumes within the cell were also analyzed, and E4 astrocytes held significantly more lipid volume under lipid loaded conditions compared to E3. (Figure 1C). Furthermore, expression of perilipin-2 (PLIN-2), a LD associated protein, was increased in E4 astrocytes in an FA-rich environment compared to E3. (Figure 1D,E and Figure S1). 

**Figure 2:**
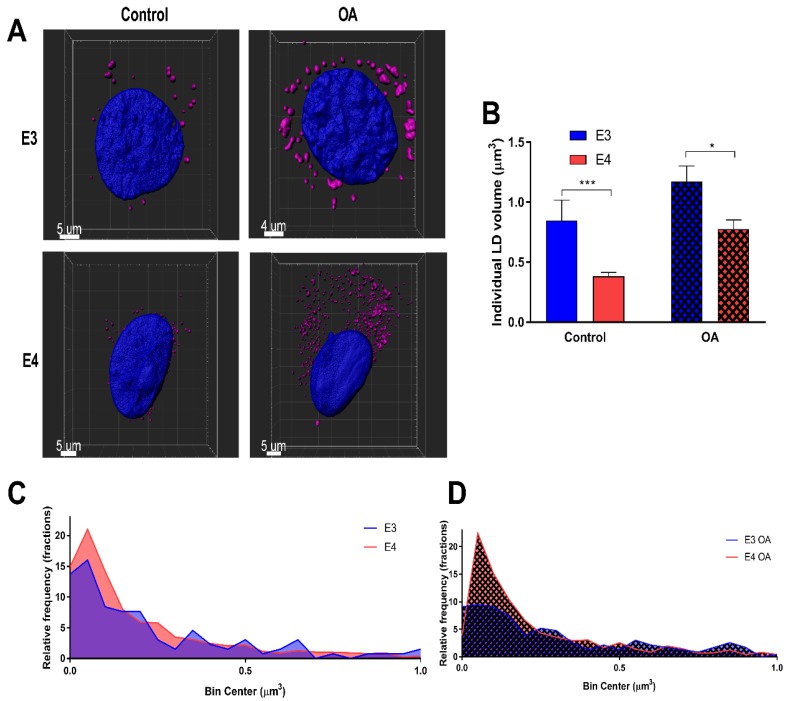
E4 astrocytes have smaller lipid droplets. (**A**) Representative 3D renderings of E3 and E4 astrocytes stained with LipidSpot and DAPI under control and lipid-loaded conditions. (**B**) Individual lipid droplet (LD) volume was quantified under control and lipid-loaded conditions. Values represented are means +/− SEM. Data were analyzed by unpaired *t*-test; *** *p* < 0.001, * *p* < 0.05. (**C**) Relative frequencies of LD volumes were binned between 0 and 1 µm^3^ under control and (**D**) lipid loaded conditions. Bin size = 0.1 µm^3^. E3 control LDs (*n* = 131); E4 control LDs (*n* = 775); E3 OA LDs (*n* = 231); E4 OA LDs (*n* = 1208).

To determine LD size and distribution, LDs in individual cells were rendered using Imaris software under control and lipid-loaded conditions (Figure 2A), and volumes were quantified (Figure 2B–D) and binned using 0.1 µm as a bin size. It was found that E4 astrocyte LDs were on average 54.8% smaller on a volume per droplet basis than E3s under control conditions, and 33.7% smaller when lipid-loaded. Together, these results suggest that E4 astrocytes form more LDs than E3 astrocytes regardless of FA exposure, as reflected in increased expression of PLIN-2 and an increased LD count. 

### 3.2. E4 Astrocytes Take Up Less Palmitate

**Figure 3:**
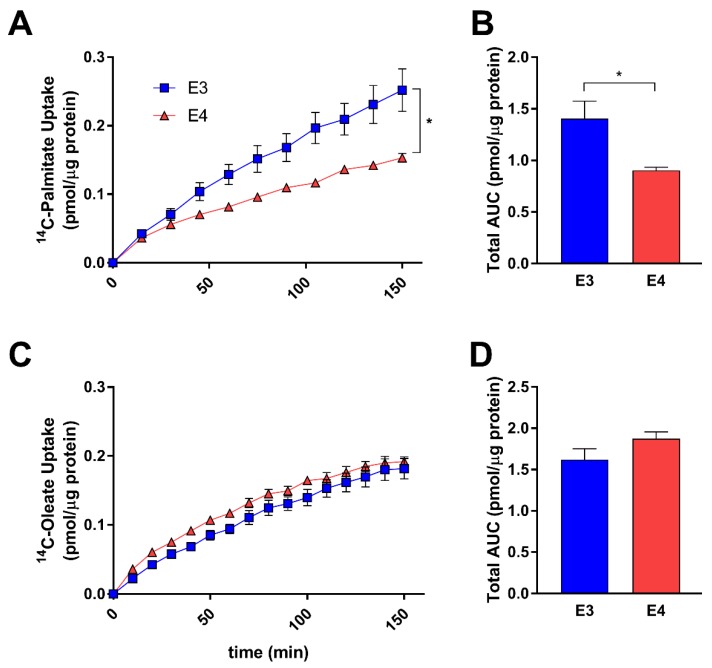
E4 astrocytes uptake less palmitate, but not oleate. E3 and E4 astrocytes were treated with 0.5 µCi/mL [1-^14^C] palmitate (**A**,**B**) or (**C**) [1-^14^C] oleate (**C**,**D**) and uptake was measured over 150 min by a scintillation proximity assay. Values represent mean +/− SEM (*n* = 6). Data were analyzed by two-way ANOVA of repeated measures (**A**,**C**) or *t*-test (**B**,**D**); * *p* < 0.05. Total area under the curve (AUC) was determined for palmitate (**B**) and oleate (**D**).

Hypothesizing that an increase in lipid storage may be a result of increased uptake of FAs, we next sought to determine if APOE regulates astrocyte FA uptake. Using a scintillation proximity assay and treating with ^14^C-labeled FAs, we found E4 astrocytes to take up significantly less palmitate (Figure 3A,B), with no significant difference in oleate uptake (Figure 3C). Conversely, there was a trend toward increased oleate uptake in E4 astrocytes, with cumulative uptake slightly higher when expressed as the area under curve despite lacking statistical significance. (Figure 3D) These data suggest that *APOE* isoforms may modulate FA uptake in astrocytes dependent on the FA species. 

### 3.3. E4 Astrocytes Oxidize Less Exogenous Fatty Acids

**Figure 4:**
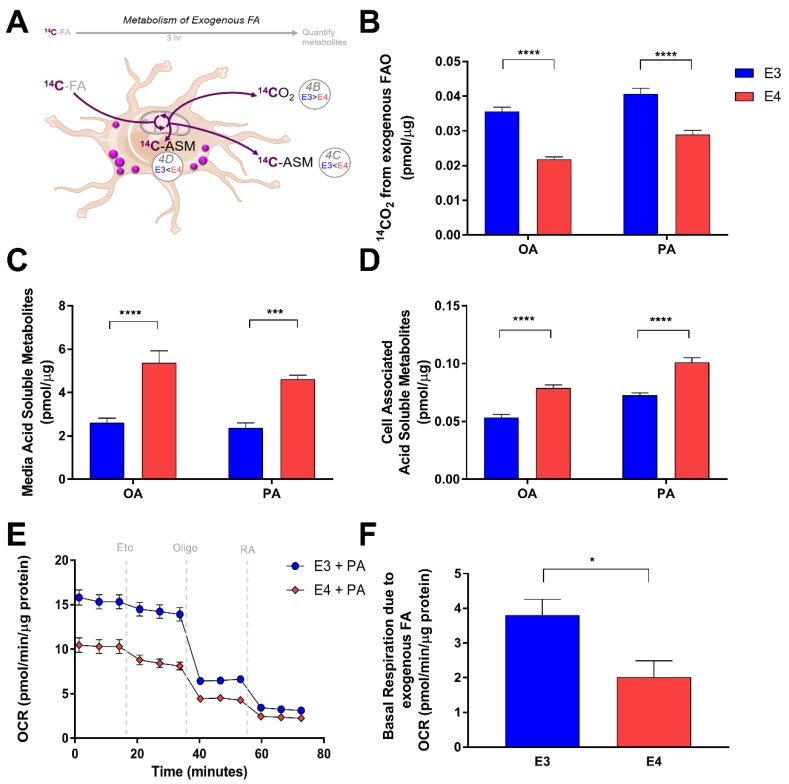
E4 astrocytes oxidize less exogenously supplied fatty acids. (**A**) Schematic and summary of pulse experiment to determine oxidation of exogenously supplied fatty acids. (**B**) E3 and E4 astrocytes were treated with [1-^14^C] oleate (OA) or [1-^14^C] palmitate (PA) and oxidation was measured over 3 h by a CO_2_ trap assay. Acid soluble metabolites were quantified in media (**C**) and intracellular (**D**) fractions. Values represent mean +/− SEM (*n* = 6). Data were analyzed by *t*-test;*** *p* < 0.001, **** *p* < 0.0001. (**E**) E3 and E4 astrocytes were pretreated for 1 h with palmitate and subjected to a Seahorse Extracellular Flux Fatty Acid Oxidation Assay. Vertical gray dashes indicate time points of pharmacological injections of etomoxir (Eto), oligomycin (Oligo), or Rotenone and Antimycin (RA). (**F**) Oxygen consumption due to FA oxidation of exogenously supplied palmitate was determined (see methods) and graphed. Values represent means +/− SEM (*n* = 3–4). Data were analyzed by unpaired *t*-test; * *p* < 0.05.

We next examined how APOE might regulate FA oxidation in astrocytes. We first assessed how E3 and E4 astrocytes oxidize exogenously supplied FA. We treated cells with radiolabeled palmitate or oleate for 3 h in a nutrient depleted media, and captured the oxidation product CO_2_, as well as incompletely oxidized products of FA metabolism in the form of acid soluble metabolites (ASM). (Figure 4A). We found that E4 astrocytes oxidize significantly less exogenously supplied oleate and palmitate than E3 astrocytes (Figure 4B), and instead accumulate significantly more metabolites that are acid soluble (Figure 4C,D); these ASM include ketone bodies, acyl carnitines, citric acid cycle intermediates, and FA less than six carbons in length [36]. We found an increased concentration of these ASM inside E4 cells (Figure 4C). We also found an increased concentration of ASM in the media (Figure 4D), suggesting that E4 astrocytes may export more FA metabolites than E3s. Using Seahorse Extracellular Flux Analysis, we then measured the oxygen consumption rate (OCR) before and after inhibition of CPT-1 in E3 and E4 astrocytes supplied with palmitate in the media (Figure 4E). We found E4 astrocytes to exhibit a lower OCR compared to E3 at all time points (Figure 4E). This lower OCR is likely primarily due to a decreased rate of glucose oxidation, as E4 is strongly associated with a phenotype of glucose hypometabolism [4,37]. When we specifically calculated the contribution of FA oxidation to OCR by comparing basal OCR in palmitate treated versus BSA treated astrocytes, we found that E4 astrocytes utilize significantly less exogenously supplied FA as part of their basal respiration (Figure 4F).

**Figure 5:**
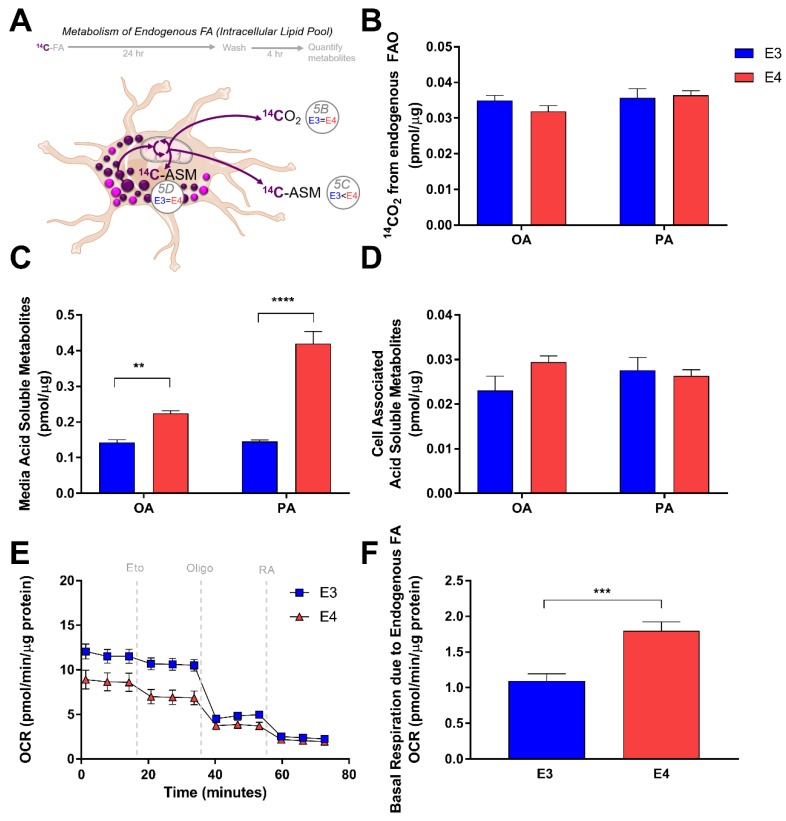
E4 astrocytes oxidize more endogenous fatty acids. (**A**) Schematic and time-course of pulse-chase experiment to determine oxidation of endogenous fatty acids.. (**B**) E3 and E4 astrocytes were treated with 1 µCi/mL [1-^14^C] oleate (OA) or [1-^14^C] palmitate (PA) combined with non-labelled isotopes to 200 µM in Advanced DMEM for 24 h, washed with PBS, and then oxidation was measured over 4 h in a nutrient depleted media by a CO_2_ trap assay. Acid soluble metabolites were quantified in media (**C**) and intracellular (**D**) fractions. Values represent mean +/− SEM (*n* = 6). Data were analyzed by *t*-test; ** *p* < 0.01, *** *p* < 0.001; **** *p* < 0.0001. (**E**) E3 and E4 astrocytes were pretreated for 1 h with bovine serum albumin supplemented media and subjected to a Seahorse Extracellular Flux Fatty Acid Oxidation Assay. Vertical gray dashes indicate time points of pharmacological injections of etomoxir (Eto), oligomycin (Oligo), or Rotenone and Antimycin (RA). (**F**) Oxygen consumption due to intracellular FA oxidation was determined (see methods) Results were normalized to protein content. Values represent means +/− SEM (*n* = 3–4). Data were analyzed by unpaired *t*-test; *** *p* < 0.0005.

Since E4 astrocytes showed a significant increase in LD accumulation, we next studied how APOE modulates FA oxidation from intracellular FA pools. Knowing that incubation with oleate for 24 h induces LD formation, we “pulsed” E3 and E4 astrocytes with radiolabeled FAs for 24 h to incorporate the radiolabel into LDs (Figure 5A). We then aspirated the media, washed with PBS, and captured oxidation and non-oxidation products of the intracellular FAs after a 4 h “chase”. Interestingly, in contrast to the oxidation of exogenously supplied FA, E3 and E4 astrocytes showed no difference in CO_2_ production from intracellular pools of oleate or palimate (Figure 5B). Quantification of ASMs of intracellular palmitate and oleate revealed that E4 astrocytes once again exported significantly more ASM into the media (Figure 5C), and have similar levels within the cells (Figure 5D). We next quantified OCR using Seahorse in E3 and E4 astrocytes that were incubated in serum free media and not supplied with exogenous FA (Figure 5E), assuming the only source of FA would be from intracellular LDs. We found that the OCR in E4 astrocytes that could be attributed to oxidation of endogenous FA was significantly higher than E3 astrocytes, suggesting that while CO_2_ production is similar between genotypes, E4 astrocytes seem to consume more oxygen from the metabolism of intracellular FAs (Figure 5F). Together these data suggest that APOE alters FA oxidation in astrocytes, and that these effects differ depending on the source of FAs (endogenously stored as LD vs exogenously supplied in media). 

### 3.4. E4 Astrocytes Are More Sensitive to CPT-1 Inhibition

**Figure 6:**
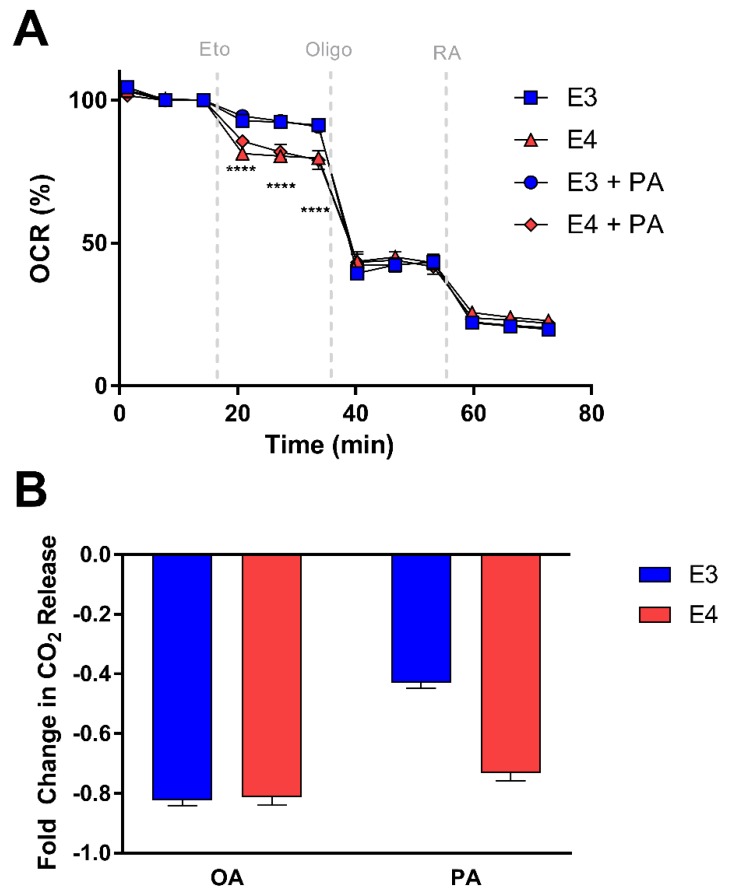
E4 astrocytes are more sensitive to CPT-1 inhibition. (**A**) Percent change in OCR for E3 and E4 astrocytes pretreated with BSA or palmitate-BSA. Data represent mean of replicate wells +/− SEM (*n* = 3–4). Data were analyzed by two-way ANOVA, repeated measures; **** *p* < 0.0001. (**B**) Fold change in oxidation of oleate (OA) or (PA) due to carnitine palmitoyltransferase 1 (CPT-1) inhibition was determined by CO_2_ trap assays and represented as mean of replicate wells +/− SEM (OA: *n* = 4; PA: *n* = 3). Data were analyzed by *t*-test; **** *p* < 0.0001.

Since CPT-1 is the rate-limiting enzyme of fatty acid oxidation, we hypothesized that E3 and E4 astrocytes may exhibit varying responsiveness to CPT-1 inhibition. Using Seahorse, we measured OCR before and after CPT-1 inhibition with etomoxir and assessed the percent change in OCR from baseline in E3 and E4 astrocytes pretreated with palmitate and the non-pretreated control cells (Figure 6A). We found that E4 astrocytes have a significantly larger drop in OCR after CPT-1 inhibition compared to E3 astrocytes in both pretreated and control cells, suggesting an increased reliance on FA oxidation for overall OCR (Figure 6A). 

To determine if CPT-1 inhibition results in APOE genotype specific effects on palmitate and oleate oxidation, we used a ^14^CO_2_ trap approach and found E4 astrocytes to exhibit a greater fold change in oxidation of palmitate from the baseline after CPT-1 inhibition (Figure 6B). However, we saw no differences between genotypes with regards to the etomoxir effect on oleate oxidation (Figure 6B), again suggesting these APOE effects may be specific to certain FA species.

## 4. Discussion

Lipid droplets in astrocytes serve to both sequester fatty acids that would otherwise be cytotoxic if free in the cytoplasm and to offer an energy rich and accessible pool for cellular metabolic needs in times of starvation and stress. Recent studies of AD brain tissue have demonstrated an increase in LDs [24,25]. ApoE is an important component of peripheral and central nervous system lipoproteins, which share many biochemical features with LDs [19]. The E4 allele of *APOE* is a critical genetic risk factor for late onset AD, and is strongly associated with a number of metabolic abnormalities [4,29,38,39]. However, the link between E4 and LD biology has not been widely explored. In the current study, we characterized a number of apoE isoform specific differences in LD formation and FA utilization in astrocytes.

We found that E4 astrocytes form many small LDs compared to the few large LDs observed in E3 astrocytes. The structural makeup of LDs could be a contributor to overall LD size. Surfactant lipids such as phosphotidylcholine (PC) are known to stabilize LD emulsions, while more fusogenic lipids, such as phosphatidic acid, promote LD coalescence [19,40]. Our group previously showed that E4 mice brains differ in various lipid concentrations using an unbiased metabolomics approach [41]. Interestingly, Heinsinger et al. recently showed that the apoE isoforms are associated with varying sizes of CNS lipoproteins, with E4 individuals and astrocytes synthesizing smaller apoE-associated lipoprotein particles [42]. As the structure and makeup of LDs and lipoproteins are similar, it is possible that E4 promotes smaller spherical lipid carriers whether that be inside or outside the cell. Overall isoform specific effects on FA metabolism could also be a result of differing total concentrations of apoE. E4 astrocytes are known to secrete less apoE into the media and have less intracellular apoE (Figure S2) [33], and E4 brains from targeted replacement mice have less apoE [43]. Whether the lower quantity of apoE4 in E4 carriers is related to disease pathogenesis is unknown. 

LD associated proteins, such as perilipins, serve to stabilize the LD from excessive lipolysis. Loss of perilipin-2 has been associated with an increase in endogenous fatty acid oxidation [44]. We found that E4 astrocytes exhibit increased expression of perilipin-2, decreased exogenous FA oxidation, and increased oxygen consumption from endogenous fatty acid oxidation. Overexpression of PLIN2 has been liked to a decrease in glucose uptake by LD sequestration of key regulators of glucose transporters [45]. In unpublished work, our group found that E4 down-regulates astrocyte glucose metabolism, much like the effects reported in neuronal models of apoE overexpression [38]. It may be that the energetic needs of an E4 astrocyte in the absence of robust glucose metabolism overpower the inhibitory effect of PLIN2 on LD lipolysis. The overexpression of PLIN2 has also been shown to increase LD formation in various cell types [46,47,48]. This is congruent with our findings in regards to E4 astrocytes, where an increase in LD number paralleled with increased PLIN2 expression. In hepatocytes, increased levels of reactive oxygen species (ROS) prompt increased *PLIN2* expression subsequent LD formation [46]. ROS has been linked to LDs in glia in a Drosophila model as well, and this process was found to be apoE dependent [27,49]. Oxidative stress as an inducer of LDs in astrocytes as a function of *APOE* deserves further investigation in light of the poor anti-oxidant capacity of E4 [50]. Alternatively, the presence of many small droplets as opposed to larger droplets could be a result of increased FA oxidation resulting from LD turnover. More studies are needed in CNS in vitro models to define LD metabolism and how it may differ from peripheral LD metabolism. 

E4 carriage in humans seems to promote an “accelerated AD” status, particularly in terms of cerebral glucose metabolism [4]. Namely, as individuals transition through mild cognitive impairment to AD, glucose metabolism progressively decreases in several distinct brain regions [51,52]. E4 individuals, however, show a state of glucose hypometabolism in these same regions even in the absence of cognitive impairment, and as early as the third decade of life [7]. LDs are known to increase in cerebral density with age and are found in GFAP+ cells [53]. E4 carriers show an accelerated development of AD neuropathology and cognitive symptoms, and astrocytes have been shown to play a key role in E4-driven AD pathogenesis [54]. Perhaps this accelerated AD phenotype in E4 individuals extends to cerebral LD accumulation as well. 

It has been proposed that E4 carriers may be at risk for cognitive decline because of an E4-driven disruption in FA metabolism [55,56]. Here we showed that APOE regulates fatty acid metabolism in the astrocyte depending on the saturation status of the fatty acid. We observed marked decrease in uptake of the fully saturated fatty acid palmitate in E4 astrocytes compared to E3 astrocytes while no difference was observed in oleate uptake. When we treated with etomoxir, we saw drastic reduction in oleate oxidation in both genotypes, but much less of a reduction in oxidation in E3 astrocyte palmitate oxidation compared to E4. These data may shed light on a phenomenon seen in epidemiological studies where E4 carriers respond well to an acute high fat meal (by cognitive measures and plasma AD biomarkers), while non-E4 carriers responded poorly [57]. This may be due to a lower uptake of saturated fatty acids. Conversely, E4 carriers have been shown to exhibit increased uptake of unsaturated fatty acids, specifically docosahexaenoic acid [58]. This could be related to the preventative effect of a Nordic diet rich in fish, fruits, and vegetables on cognitive decline in E4 individuals [59]. Indeed, apoE isoform specific effects on lipid usage have been characterized systemically, wherein E4 mice show a metabolic preference for oxidizing FAs [60]. Related FA disturbances have been characterized in humans carrying E4. For instance, E4 carriers show greater uptake, brain incorporation, and whole-body β-oxidation of docosahexaenoic acid compared to non-E4 carriers [58,61]. In the current study, our data using ^14^C-labeled FAs support the idea that E4 alters the flux of metabolites into the TCA cycle and because of this, decreases CO_2_ production and increases metabolites that are acid soluble. Our group previously showed that E4 alters numerous pathways within the cerebral metabolome [41], and others have shown changes in TCA specific metabolites in the brain [62]. 

## 5. Conclusions

In this study we showed that E4 astrocytes display an increase in LD formation compared to E3 astrocytes. Specifically, E4 astrocytes have a higher number of smaller LD and increased expression of the LD marker PLIN2. We also observed increased oxygen consumption rates from endogenous fatty acid oxidation, decreased rates of exogenous fatty acid oxidation, and an increased sensitivity to CPT-1 inhibition in E4, compared to E3, astrocytes. While a number of important questions remain, we hope these initial findings will serve as a basis for the further study of APOE-associated alterations in astrocyte metabolism and future treatments of AD and other neurodegenerative diseases. 

## Supplementary Materials

The following are available online at http://www.mdpi.com/2073-4409/8/2/182/s1, Figure S1: E4 astrocytes express more perilipin-2, Figure S2: E4 astrocytes secrete less ApoE into the media and have less intracellular ApoE, Figure S3: E4 astrocytes form more lipid droplets.
