# Supplementary material for: Apolipoprotein E4 Alters Astrocyte Fatty Acid Metabolism and Lipid Droplet Formation
Source: Cells. 2019 Feb 20;8(2):182. doi: 10.3390/cells8020182 (PMC6406677; doi:10.3390/cells8020182)
Supplement: Supplementary file 1 [file cells-08-00182-s001.pdf]

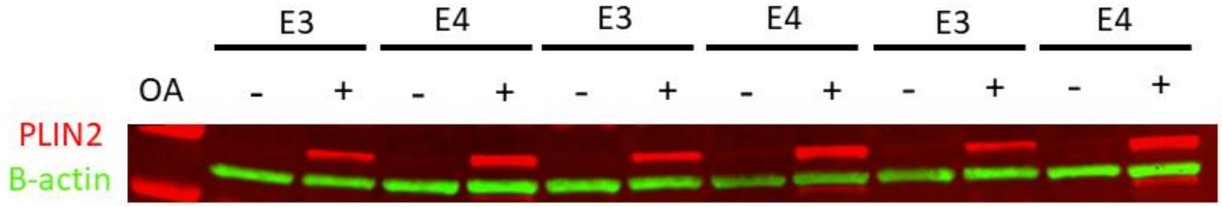

Figure S1. E4 astrocytes express more perilipin-2. E3 and E4 astrocytes were incubated in Advanced DMEM with or without 250 µM oleic acid (OA) conjugated to BSA. Protein was extracted by RIPA lysis and 20µg was loaded for immunoblot analysis of perilipin-2 (PLIN2with β-actin as a loading control.

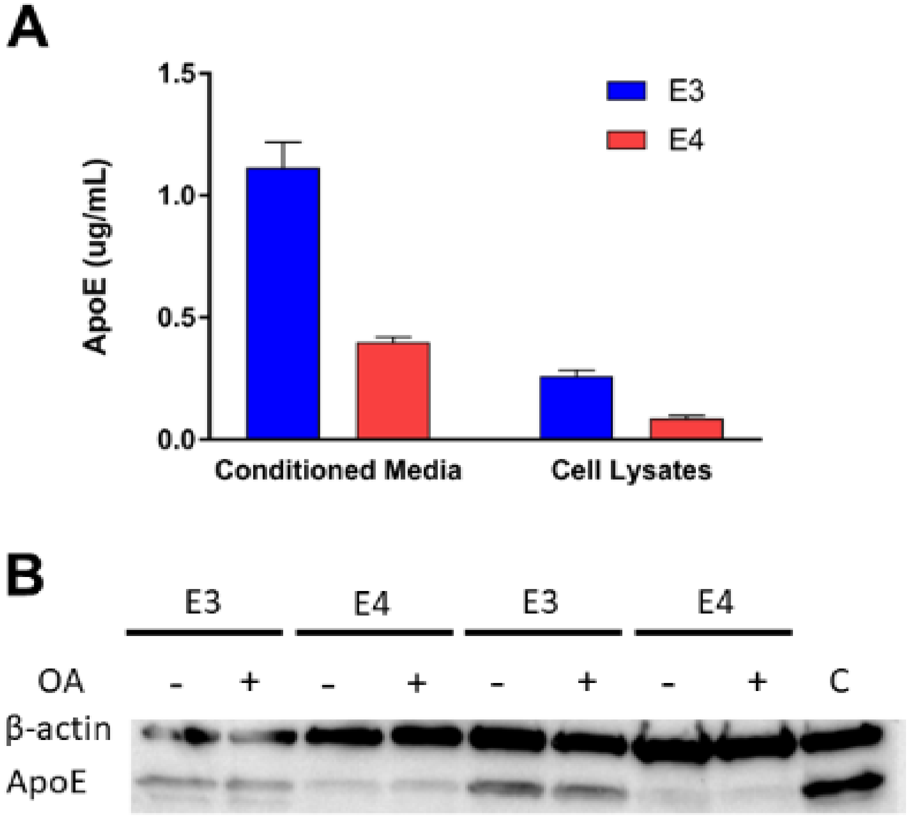

Figure S2. E4 astrocytes secrete less ApoE into the media and have less intracellular ApoE. (**A**) Media and cell lysates from E3 and E4 astrocyte cell cultures were isolated. Samples were assayed for ApoE using Abcam Human ApoE ELISA kit as previously described [1]. (**B**) E3 and E4 cell lysates were separated using SDS-PAGE and immunoblotted for total human ApoE and β-actin as loading control.

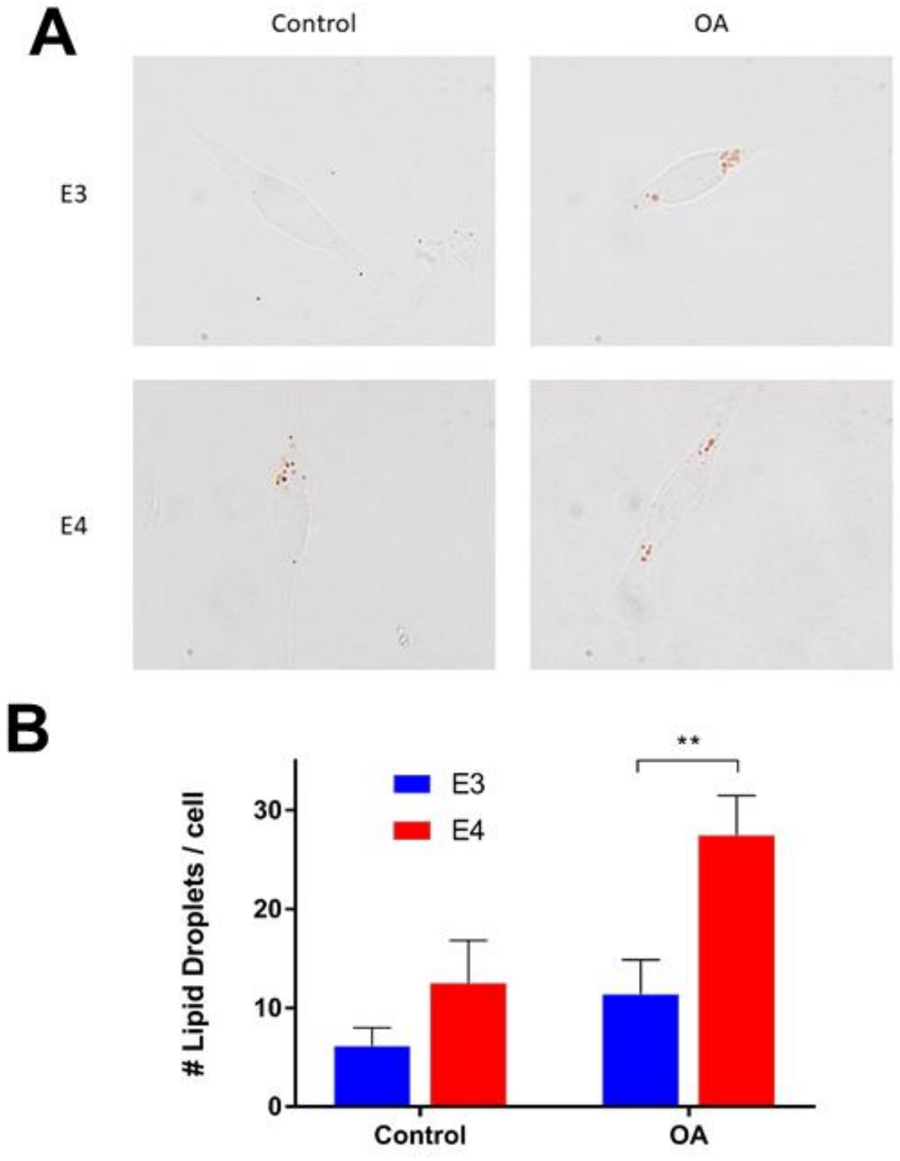

Figure S3. E4 astrocytes form more lipid droplets. (**A**) E3 and E4 expressing astrocytes were lipid loaded for 24 hours in control or oleic acid supplemented media. Cells were fixed and incubated with oil red O to stain lipid droplets. (**B**) Oil red O stained LDs were quantified using image J. Values represent means +/- SEM from 8 images. Data was analyzed by t-test. **$p<0.005$

Supplementary Methods

Oil Red O Histology

Oil Red O staining was performed as previously described [1]. Astrocytes were plated on TissueTek chamber slides. After lipid incubation, media was aspirated and slides were washed 2X with sterile PBS. 4% PFA was added for 30 min at 37 C to fix the cells, followed by a PBS wash. 60% isopropanol was added to the chamber wells for 5 min for permeabilization. Isopropanol was aspirated and the cells were

dried. Oil red O was then added to the chamber wells for 20 min followed by 3 washes in ddH_2O. Chambers were removed from the slides and then coverslips were mounted. Images were acquired at 100X on a phase contrast Nikon microscope under oil immersion.

1. Johnson, L.A.; Arbones-Mainar, J.M.; Fox, R.G.; Pendse, A.A.; Altenburg, M.K.; Kim, H.-S.; Maeda, N. Apolipoprotein e4 exaggerates diabetic dyslipidemia and atherosclerosis in mice lacking the ldl receptor. *Diabetes* **2011**, *60*, 2285–2294.
